# TSNAD: an integrated software for cancer somatic mutation and tumour-specific neoantigen detection

**DOI:** 10.1098/rsos.170050

**Published:** 2017-04-05

**Authors:** Zhan Zhou, Xingzheng Lyu, Jingcheng Wu, Xiaoyue Yang, Shanshan Wu, Jie Zhou, Xun Gu, Zhixi Su, Shuqing Chen

**Affiliations:** 1 Zhejiang Provincial Key Laboratory of Anti-Cancer Drug Research, College of Pharmaceutical Sciences, Zhejiang University, Hangzhou 310058, People's Republic of China; 2 College of Computer Science and Technology, Zhejiang University, Hangzhou 310013, People's Republic of China; 3 Department of Genetics, Development and Cell Biology, Program of Bioinformatics and Computational Biology, Iowa State University, Ames, IA 50010, USA; 4 State Key Laboratory of Genetic Engineering and MOE Key Laboratory of Contemporary Anthropology, School of Life Sciences, Fudan University, Shanghai 200438, People's Republic of China

**Keywords:** cancer somatic mutation, tumour antigen, neoantigen, major histocompatibility complex, membrane protein

## Abstract

Tumour antigens have attracted much attention because of their importance to cancer diagnosis, prognosis and targeted therapy. With the development of cancer genomics, the identification of tumour-specific neoantigens became possible, which is a crucial step for cancer immunotherapy. In this study, we developed software called the tumour-specific neoantigen detector for detecting cancer somatic mutations following the best practices of the genome analysis toolkit and predicting potential tumour-specific neoantigens, which could be either extracellular mutations of membrane proteins or mutated peptides presented by class I major histocompatibility complex molecules. This pipeline was beneficial to the biologist with little programmatic background. We also applied the software to the somatic mutations from the International Cancer Genome Consortium database to predict numerous potential tumour-specific neoantigens. This software is freely available from https://github.com/jiujiezz/tsnad.

## 1. Introduction

Tumour antigens have attracted much attention for their importance in cancer diagnosis, prognosis and targeted therapy, as they are crucial tumour biomarkers for identifying tumour cells and are potential targets for cancer therapy [1–3]. Tumour antigens can be broadly classified into two categories based on their specificity: tumour-specific antigens, which are only present in tumour cells; and tumour-associated antigens, which are overexpressed or aberrantly expressed in tumour cells and are also expressed in some normal cells [1]. In addition to abnormal expression patterns, tumour cells also contain a range of cancer somatic mutations and mutations in protein-coding regions might produce tumour-specific mutant proteins [4,5]. Tumour antigens derived from these tumour-specific mutant proteins are unparalleled tumour biomarkers, as they are only produced by tumour cells and are potential tumour-specific mutant antigens or neoantigens [3].

Tumour antigens recognized by T cells or antibodies should present on the surface of tumour cells [6,7]. A major part of tumour antigens used as drug targets are membrane proteins, such as HER2 and CD19, which are targets of the antibody trastuzumab [8] and chimaeric antigen receptor T-cell immunotherapy (CAR-T) for B-cell cancer [9,10], respectively. Additionally, tumour antigens presented by class I major histocompatibility complex (MHC) molecules for recognition by T cells (i.e. tumour-specific neoantigens) could also be used as drug targets [2,11,12]. On the other hand, in the immune checkpoint blockade therapy, the neoantigen load is associated with the therapy efficacy (i.e. PD-1, CTLA-4 blockade), which indicates that the neoantigen load is a great biomarker in cancer immunotherapy [13]. Because of their potential application to be targets and biomarkers in cancer immunotherapy [1,12,14,15], tumour-specific neoantigens have attracted much attention in biomedical research. Several prediction tools have been developed to predict tumour-specific neoantigens from cancer somatic mutations, such as pVAC-seq [16] and INTEGRATE-neo [17], which can predict neoantigens produced by non-synonymous somatic mutations and gene fusions, respectively. However, these tools only predict neoantigens presented by class I MHC molecules that can be recognized by T cells, they do not consider the mutations in the extracellular regions of membrane proteins that can be recognized by mutation-specific antibodies [18,19].

In this study, we developed integrated software with a graphical user interface (GUI), called the tumour-specific neoantigen detector (TSNAD), which can identify cancer somatic mutations following the best practices of the genome analysis toolkit (GATK v. 3.5) [20] from the genome/exome sequencing data of tumour-normal pairs. We also provided a filter for calling tumour-specific mutant proteins. Then, we conducted two strategies to predict neoantigens. First, we extracted the extracellular mutations of membrane proteins according to the protein topology. Second, we invoked NetMHCpan (v. 2.8) [21] to predict the binding information of mutant peptides to class I MHC molecules. Finally, we applied TSNAD on the cancer somatic mutations collected in the International Cancer Genome Consortium (ICGC) database to predict potential neoantigens.

## 2. Material and methods

### 2.1. Tools

Standard sequencing data processing consists of preprocessing, alignment, variants calling, annotation and further analysis. Given that the existing software or tools are designed for specific functions, it was necessary to develop an automated and user-friendly framework that calls a series of software. This section summarizes the required software and its main features.

#### 2.1.1. Data filtering software

Trimmomatic (v. 0.35) [22]. Original raw sequences have random lengths and contain adaptors that will be harmful to the subsequent data processing. This software can trim and crop raw reads and remove artefacts.

#### 2.1.2. Genome mapping software

Burrows-Wheeler Aligner (BWA, v. 0.7.12) [23,24]. This alignment toolkit is used for mapping short sequences to a reference genome. This software is based on the Burrows-Wheeler transformation and is highly efficient at finding locations of low-divergent sequences on a large genome.

#### 2.1.3. Alignment manipulating tool

Samtools (v. 1.3) [25]. Its view and sort functions transform sequencing data format from SAM (sequence alignment/map) to BAM (binary alignment/map), which will save an enormous amount of storage space. Moreover, it can manage duplicate reads and index alignments.

#### 2.1.4. Data processing tool

Picard tools (v. 1.140) [26]. This program consists of a set of Java command lines to handle with different sequencing data format (such as SAM, BAM and VCF). Given redundancy data may influence further processing, Picard MarkDuplicates tool can thus be applied to remove repeat sequences.

#### 2.1.5. Variant calling software

Genome Analysis Toolkit (GATK v. 3.5) [20], Mutect2 [27]. The main function of GATK is variant discovery in high-throughput sequencing data. Mutect2 is a package in GATK to identify somatic SNVs and INDELs.

#### 2.1.6. Mutation annotation software

Annovar (14 December 2015) [28,29]. We use it to functional annotate somatic mutations, including position, change of nucleotide, change of amino acid for protein-coding region, and other functions. We can then extract tumour-specific mutant proteins.

#### 2.1.7. Human leucocyte antigen typing software

SOAP-HLA (v. 2.2) [30]. This software detects the human leucocyte antigen (HLA—the MHC in humans) types for each sample. The program takes sorted aligned sequencing data (BAM format) as the input and outputs HLA types. The HLA types are critical for the MHC-binding predictions.

#### 2.1.8. Protein topology indicating software

TMHMM (v. 2.0) [31]. This tool is used to predict the topology of membrane proteins based on a hidden Markov model (HMM). The prediction of transmembrane helices and membrane proteins is highly accurate [32].

#### 2.1.9. Major histocompatibility complex-binding predicting software

NetMHCpan (v. 2.8) [21]. This software can forecast peptides that can bind to MHC class I molecules using artificial neural networks.

### 2.2. Datasets

The somatic mutations were collected from the whole-genome/exome sequencing data of 9155 tumour-normal pairs in the ICGC database (Release 20, http://icgc.org). This dataset has compiled over 1.5 million sample somatic mutations in coding regions, among which 828 129 missense variants have caused amino acid changes with a frequency range from 1 to 476 out of 9155 tumour samples.

The HLA types were extracted from the 1000 Genome Project. We choose 16 HLA alleles with frequencies of more than 5% in the population collected in the 1000 Genome Project [33], which includes five HLA-A (HLA-A*01:01, HLA-A*02:01, HLA-A*03:01, HLA-A*11:01 and HLA-A*24:02), four HLA-B (HLA-B*07:02, HLA-B*35:01, HLA-B*40:01 and HLA-B*51:01) and seven HLA-C (HLA-C*01:02, HLA-C*03:03, HLA-C*03:04, HLA-C*04:01, HLA-C*06:02, HLA-C*07:01 and HLA-C*07:02) alleles.

### 2.3. Identification of extracellular region of membrane proteins

The list of human membrane proteins was extracted from the human protein atlas [34]. The amino acid sequences of these membrane proteins were downloaded from Ensembl (GRCh37 v. 75) [35]. TMHMM (v. 2.0) was used to identify the transmembrane topology and extracellular region of each membrane protein [31].

### 2.4. Prediction of class I major histocompatibility complex binding

**Figure 1.**
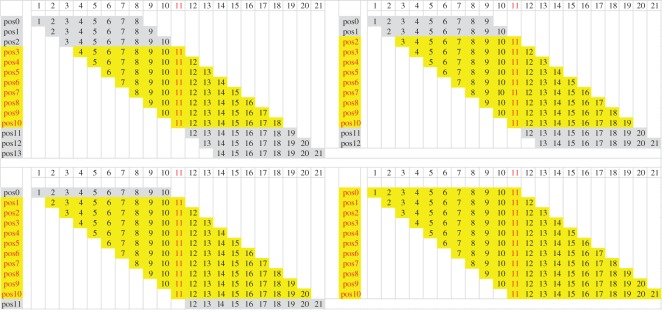
Mutant peptides with 21 amino acids and corresponding 8–11 mer peptides. MHC molecules always bind to 8–11 mer peptides, so we extracted peptides 21 amino acids in length, with 10 amino acids upstream and 10 amino acids downstream of mutation sites for NetMHCpan prediction. The number 11 in red indicates the mutated site, and the peptides in yellow represent all the possible peptides which may bind to MHC molecules.

After we obtained the list of the tumour-specific mutant proteins, we extracted the peptide sequences around the mutation sites. As MHC molecules always bind to peptides 8–11 amino acids in length, we extracted peptides 21 amino acids in length, with 10 amino acids upstream and 10 amino acids downstream of mutation sites for NetMHCpan prediction (figure 1). Wild-type peptides with the same length as the mutant peptides were extracted as references. These wild-type and mutant peptides were measured for their binding affinities (50% inhibitory concentration [IC_50_], nM) to each class I HLA allele. The binding was considered strong if the IC_50_ value was less than 150 nM, and a weak binding had an IC_50_ value between 150 and 500 nM. Non-binding occurred if the IC_50_ value was more than 500 nM [11].

### 2.5. Experimental validation of peptide biding to class I major histocompatibility complex molecular

Peptides were obtained lyophilized (more than 95% purity) from Bankpeptide Biological Technology Co., Ltd (Hefei, China), dissolved in 10% DMSO in sterile water and tested for sterility, purity, endotoxin and residual organics. Peptide binding to HLA-A*02:01 was determined by T2 assay [36]. T2 cells were washed in phosphate buffered saline (PBS) and RPMI-1640 without serum. In total, 5 × 10^5^ cell ml^–1^ were incubated with 5 µg ml^−1^ peptide and 10 µg ml^−1^ human beta-2-microglobulin in serum-free RPMI-1640 for 4 h or overnight at 37°C. The pulsed cells were pelleted and followed by 3 × 1 ml rinses in PBS with centrifugation at 500*g* for 5 min at 4°C. Cells were resuspended in 200 µl PBS and stained with 1 µl of w6/32 (Thermo Fisher) for 30 min on ice, followed by three rinses with 1 ml PBS at 4°C. Cells were then resuspended in 200 µl PBS and 1 µl of goat anti-mouse antibody-FITC (Beyotime Biotechnology) for 30 min on ice, followed by three rinses at 4°C. Then, cells were resuspended in 500 µl PBS. Stained T2 cells were analysed using a FACSCalibur.

## 3. Results

### 3.1. Software overview

We developed integrated software, called TSNAD, under the Linux operation system through a GUI. The platform is completely automated and is mainly designed for users who have little programming experience. There are several neoantigen prediction pipelines such as pVAC-seq, INTEGRATE-neo: pVAC-seq combined the tumour mutation and expression data to predict neoantigens by invoking NetMHC v. 3.4; INTEGRATE-neo was designed to predict neoantigens from fusion genes based on the pipeline INTERGRATE and NetMHC v. 4.0. Similar with these pipelines, TSNAD also used widely approved software NetMHCpan v. 2.8 to predict neoantigens. Compared with other neoantigen prediction pipelines, TSNAD has lists of advantages: first, TSNAD offered a pipeline for mutation calling from sequencing data; second, TSNAD not only considered the neoantigens presented by class I MHC molecules, but also took mutations in membrane proteins into consideration; third, unlike other pipelines that performed through command lines, TSNAD provided a GUI for biologists without programming background to analyse their data easily. The software consists of two toolkits: mutation detection and neoantigen prediction. Each toolkit is a two-step process as follows: configure the parameters and run the corresponding toolkit.

The first step is to configure the software paths and parameters. This step is of great significance, and users are expected to ensure the appropriateness and correctness of the configurations. Users can find the detailed instructions about how to set paths and parameters in the user's manual. For the software paths, the users do not need to change these parameters once they are set because TSNAD will import the existing configuration files by default. Users can also edit partial parameters by GUI or by manually modifying the configuration files. It is worth noting that TSNAD requires its own naming convention for the input files. The users can choose to either manually or use the tool we provided to rename the names of sequencing files to suit the criteria of TSNAD.

After setting the configurations, non-expert users can run the pipeline by just clicking on the appropriate toolbar. In the processing monitoring window, the users can observe the pipeline progression. The pipeline, which was written in Python programming language (v2.7), calls for standard third-party software and applies multiprocessing strategy to speed up the data processing.

When the pipeline is finished, all of the results will be stored in a user-specified folder. The mutation detection pipeline returns the list of somatic mutations with annotations. The neoantigen prediction pipeline returns extracellular mutations of the membrane proteins and the MHC-binding information (all in TXT format).

### 3.2. Detection of cancer somatic mutations

**Figure 2.**
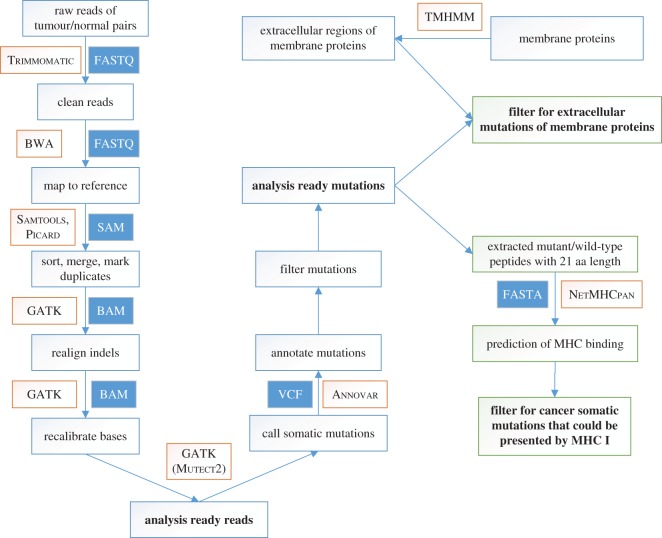
The software pipeline of TSNAD. The pipeline performs best practices for somatic SNVs and INDELs in whole-genome/exome sequence with GATK. Then, we extracted the extracellular mutations of membrane proteins according to the protein topology, and invoked NetMHCpan to predict the binding information of mutant peptides to class I MHC molecules.

The software can detect single-nucleotide variants (SNVs) and small insertions or deletions (INDELs) according to the pipeline as depicted in figure 2. The raw paired-end sequence data were in FastQ format from the whole-genome sequencing, the whole-exome sequencing or the targeted gene panel sequencing using the Illumina platform. The raw data were cleaned using Trimmomatic [22]. BWA-MEM was used to map the reads to the reference genome sequences [23,24]. Samtools [25] and Picard [26] were used to address files in SAM or BAM formats, including transform, sort, merge and mark duplicates. GATK [20] was used to pre-process the BAM files, such as realigning the INDELs and recalibrating the bases. Mutect2 [27] in GATK was used to call the somatic SNVs and INDELs between tumour and normal samples. Annovar [28,29] was used to annotate the detailed mutation information.

We further provide a filter to detect the somatic mutations in the protein-coding regions and the somatic missense variants which fit the cut-off (tumour reads > 10, normal reads > 6, tumour alteration reads > 5, variant allele frequency (VAF) in tumour DNA > 0.05 and VAF in normal DNA = 0).

### 3.3. Prediction of neoantigens

When peptides differ by only one amino acid change, specific antibodies can be generated [19,37]. Therefore, missense mutations that are present on the surfaces of tumour cells are important targets for antibody-based immunotherapy. We performed two strategies to predict the neoantigens that would present on the surfaces of tumour cells [1,2]. First, we extracted the somatic mutations in the extracellular regions of the membrane proteins. Second, we predicted the neoantigens that would present on the cell surface by evaluating the binding affinity between the peptides and class I MHC molecules.

According to the Human Protein Atlas, there were 5462 predicted membrane proteins [34]. We identified the transmembrane topologies and the extracellular regions of these proteins using TMHMM [31]. To identify the extracellular mutations of membrane proteins, the filtered cancer somatic missense variants were mapped to the extracellular regions of membrane proteins. We further verified the characteristics of the mutant amino acids. Mutations that change the polarity of the amino acids have gained more attention, as they may be more likely to cause differences in binding features to antibodies between wild-type and mutant proteins.

In addition to the membrane proteins, peptides could be present on the cell surface because of the antigen presenting system, which is mediated by MHC molecules. SOAP-HLA was used for the HLA typing of each sample [30]. NetMHCpan was used to predict the binding affinity between the class I MHC and wild-type/mutant peptides [21]. We further compared the binding information of the HLA molecules to the wild-type and mutant peptides. The mutant peptides that can bind to the HLA-A/B/C molecules were extracted for further analysis; the specific bindings of the HLA proteins to the mutant peptides were preferred for their potential to be drug targets without affecting normal tissues.

### 3.4. Prediction of neoantigens based on the somatic mutation data from the International Cancer Genome Consortium database

In previous study, we performed oncogene targeted depth sequencing on a malignant peritoneal mesothelioma [38]. Applying the TSNAD to analyse the sequence data of the tumour sample and the paired peripheral blood sample, we detected 2897 somatic SNVs and 218 somatic INDELs. Four SNVs of NOTCH2, PDE4DIP, ATP10B and NSD1 and one frameshift INDEL of BAP1 were validated by Sanger sequencing on tumour RNA. We also predicted the neoantigens on these mutated proteins, and found specific-binding of neo-peptide generated by BAP1 frameshift INDEL to HLA-B*35:42 of the patient. A polyclonal antibody of the neo-peptide of BAP1 were produced in rabbits and showed a good antibody-neoantigen specificity, which indicates that the neo-peptide of BAP1 could be a potential tumour-specific neoantigen [38].

In addition to handling original sequencing data, TSNAD could also analyse exiting mutations data to predict potential neoantigens. We applied TSNAD to the simple somatic mutations of 9155 samples from the ICGC database and predicted numerous neoantigens, including extracellular mutations of membrane proteins and peptides presented by the class I MHC molecules.

### 3.5. Prediction of neoantigens from membrane proteins

To identify the extracellular mutations of membrane proteins, we mapped all of the missense mutations to the extracellular regions of membrane proteins. A dataset containing 88 354 extracellular mutations was obtained. A majority of these extracellular mutations (89.6%, 79 198 out of 88 354) occurs only once in the 9155 donors (electronic supplementary material, table S1 and figure S1), which illustrates the high heterogeneity in tumour samples. However, membrane proteins with mutations that occur in more samples are also ideal drug targets for antibody-based immunotherapy. The top 20 frequent extracellular mutations are listed in table 1 and MUC4:H4205Q is the most frequent extracellular mutation (44 out of 9155).

**Table 1.** Top 20 most frequent extracellular mutations in 9155 donors.

Chr	Pos	ID	gene	DNA mutation	protein mutation	mutation frequency
3	195505836	MU10935	MUC4	12615C>G	H4205Q	44 out of 9155
1	29138975	MU68226	OPRD1	80G>T	C27F	25 out of 9155
1	120611960	MU869951	NOTCH2	61G>A	A21T	23 out of 9155
19	1065018	MU68245	ABCA7	6133G>T	A2045S	18 out of 9155
15	22369378	MU4380351	OR4M2	803C>T	S268F	16 out of 9155
17	37868208	MU85975	ERBB2	929C>T	S310F	15 out of 9155
3	195509676	MU586249	MUC4	8775G>C	Q2925H	15 out of 9155
7	55233043	MU589341	EGFR	1793G>T	G598 V	15 out of 9155
3	195515449	MU605883	MUC4	3002T>A	V1001E	14 out of 9155
19	9072091	MU4382243	MUC16	15355C>T	P5119S	13 out of 9155
20	17639816	MU4585427	RRBP1	1337A>C	Q446P	12 out of 9155
5	179071958	MU4110168	C5orf60	64G>C	D22H	12 out of 9155
7	146829338	MU4413315	CNTNAP2	1085G>A	G362E	12 out of 9155
1	158261127	MU4408485	CD1C	265C>T	R89C	11 out of 9155
11	5345040	MU4383907	OR51B2	488C>T	S163 L	11 out of 9155
17	21319519	MU613603	KCNJ12	865G>C	E289Q	11 out of 9155
2	46707884	MU70561	TMEM247	458A>G	Q153R	11 out of 9155
2	137814319	MU4440003	THSD7B	469G>A	E157 K	11 out of 9155
3	195511286	MU4617526	MUC4	7165G>A	D2389N	11 out of 9155
7	139167934	MU66261	KLRG2	455A>C	K152T	11 out of 9155

### 3.6. Prediction of neoantigens through major histocompatibility complex-binding information

Peptides could also present on the cell surface via the antigen presenting system, mediated by MHC class I molecules. In this manner, mutant peptides that are present exclusively in tumour cells are the potential neoantigens, and the MHC–peptide complexes are called neoantigens.

Based on the missense mutations of the 9155 tumour samples from the ICGC, we extracted peptides 21 amino acids length, with 10 amino acids upstream and 10 amino acids downstream of the mutation sites. Both the mutant and reference peptides were extracted. Combined with the 16 HLA alleles whose frequencies were more than 5% in the population collected in the 1000 Genome Project, we used our software, invoking NetMHCpan (v2.8) [21] to predict the binding affinity between HLA and the collected peptides. Then, we compared the binding information of the HLA proteins to wild-type and mutant peptides, and the specific bindings of the HLA proteins to mutant peptides were collected. These mutant peptides are seen as potential neoantigens. Finally, we obtained a dataset containing 1 420 785 records. We also analysed the distribution of the dataset (electronic supplementary material, table S2 and figure S2). The results showed a similar phenomenon with that in membrane proteins.

Mutations with more frequencies in the samples may play important roles in tumorigenesis. There are 65 potential common neoantigens whose corresponding mutations appear in at least 20 out of the 9155 donors from the ICGC database and had an IC_50_ of less than 500. The 65 neoantigens are related to the 23 somatic mutations of 12 genes (table 2; electronic supplementary material, table S3). KRAS, PIK3CA and TP53 occupy more potential neoantigens than other genes, indicating that these genes play more important roles in tumour immunotherapy, corresponding to former research results that KRAS and PIK3CA are oncogenes and that TP53 is a tumour suppressor gene [39]. Moreover, we also found some genes that have not been identified as tumour-associated genes by Cancer Gene Census also encode potential neoantigens, such as MUC4, FAM194B, OPRD1 and FRG1.

**Table 2.** Sixty five potential common neoantigens and their corresponding genes and mutation frequency.

gene	role in tumour	no. mutation	no. neoantigen
KRAS	oncogene	5	11
PIK3CA	oncogene	5	21
TP53	tumour suppressor gene	3	8
SF3B1	tumour-related gene	1	2
MUC4	—	1	1
CHEK2	tumour suppressor gene	1	2
PTEN	tumour suppressor gene	2	3
FAM194B	—	1	2
OPRD1	—	1	5
CTNNB1	oncogene	1	5
FRG1	—	1	4
GNAS	tumour-related gene	1	1

We found that the most frequent potential neoantigens are encoded by gene KRAS, which has been identified as an oncogene *in vivo*. There are six potential neoantigens related to the KRAS gene in the top 10 potential neoantigens, with two different mutations: G12D and G12 V. Among the six peptides, three of them (KLVVVGADGV, KLVVVGAVGV and KLVVVGAV) are presented by HLA-A*02:01, one (TEYKLVVVGAV) is presented by HLA-A*40:01, one (GAVGVGKSAL) is presented by HLA-A*03:04 and one (GAVGVGKSAL) is presented by HLA-C*03:03 (table 3).

**Table 3.** Top 10 neoantigens with the highest mutation frequency in 9155 donors.

gene	HLA allele	position	peptide	mutation	affinity (nM)	mutation frequency
KRAS	HLA-A*02:01	8	KLVVVGADGV	G12D	214	322 out of 9155
KRAS	HLA-A*02:01	8	KLVVVGAVGV	G12 V	112	239 out of 9155
KRAS	HLA-A*02:01	8	KLVVVGAV	G12 V	163	239 out of 9155
KRAS	HLA-B*40:01	11	TEYKLVVVGAV	G12 V	90	239 out of 9155
KRAS	HLA-C*03:04	3	GAVGVGKSAL	G12 V	172	239 out of 9155
KRAS	HLA-C*03:03	3	GAVGVGKSAL	G12 V	172	239 out of 9155
PIK3CA	HLA-C*07:02	2	ARHGGWTTKM	H1047R	218	200 out of 9155
PIK3CA	HLA-C*06:02	3	ARHGGWTTKM	H1047R	457	200 out of 9155
PIK3CA	HLA-C*07:01	2	ARHGGWTTKM	H1047R	249	200 out of 9155
PIK3CA	HLA-A*11:01	11	STRDPLSEITK	E545 K	81	182 out of 9155

**Figure 3.**
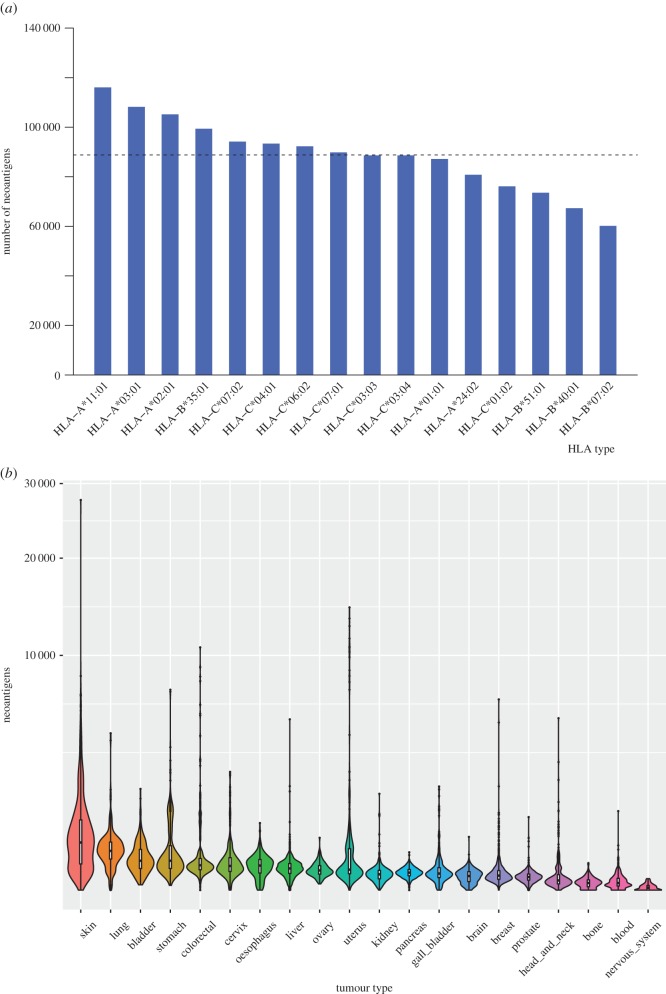
The distribution of tumour-specific neoantigens across 16 HLA types and 20 tumour types. (*a*) The number of tumour-specific neoantigens with each HLA type is shown in decreasing order. The dashed line indicates the average number of neoantigens. (*b*) Distribution of tumour-specific neoantigens across 20 tumour types. The width of each violin indicates the proportion of donors sharing a certain number of neoantigens in each tumour type. Upper limit and lower limit of white bar and the black line in it denote upper quartile, lower quartile and median number for each type.

To study the distribution of the neoantigens across different HLA type, we classified the 1 420 785 records into 16 parts according to the HLA type we used (figure 3*a*). It was found that approximately 10 mutant peptides could bind to each HLA type in each sample, which means that we can find about 60 neoantigens in each tumour sample on average.

Because of the highly heterogeneity of tumours, we further investigated the distribution of neoantigens in each tumour type (based on the tissue origin; figure 3*b*). The results showed that the neoantigen load is related to the somatic mutation burden. The cancer types have more mutation load, such as skin and lung cancer, have more neoantigens in average. Interestingly, uterus cancer has the largest number of neoantigens on average (715.98, electronic supplementary material, table S4), but the median number of neoantigens of uterus cancer ranks 10th among the 20 cancer types (figure 3*b*). The reason may be that the number of neoantigens varies greatly among different patients of uterus cancer, several uterus tumours have large numbers of neoantigens. The nervous system cancer possesses the least neoantigens (2.39) on average. The results indicated that the neoantigen load is not only quite different between different cancer types, but also quite different between different tumours from the same tissue.

### 3.7. Specific binding of the TP53 mutant peptide to HLA-A*02:01

**Figure 4.**
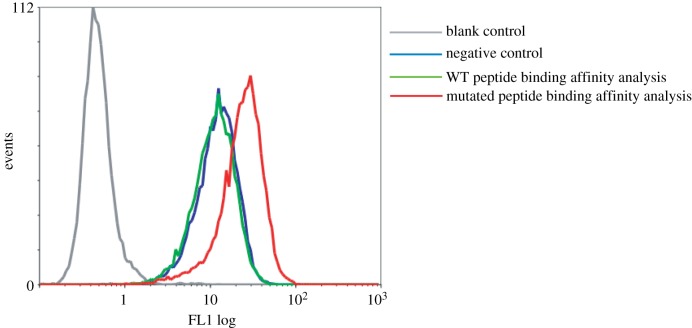
Specific binding of mutant peptide of TP53 to HLA-A*02:01. Blank control: FITC-goat anti-mouse IgG + T2 cells; negative control: human beta-2-microglobulin were incubated with T2 cells overnight at 37°C + W6/32 + FITC-goat anti-mouse IgG; wide-type (WT) peptide binding affinity analysis: WT peptide (GMNRRPILTII) and human beta-2-microglobulin were incubated with T2 cells overnight at 37°C + W6/32 + FITC-goat anti-mouse IgG; mutated peptide binding affinity analysis: mutated peptide (GMNWRPILTII) and human beta-2-microglobulin were incubated with T2 cells overnight at 37°C + W6/32 + FITC-goat anti-mouse IgG.

We choose one of the 65 potential common neoantigens, which was generated by the TP53 R248 W mutation, to experimentally confirm the specific-binding of neoantigen to HLA-A*02:01 using T2 assay [36]. We predicted that the wild-type (WT) peptide (GMN**R**RPILTII) could not bind to HLA-A*02:01, while the mutant peptide (GMN**W**RPILTII) could weakly bind to HLA-A*02:01 with the IC_50_ value = 350 nM (electronic supplementary material, table S3). The T2 cell line was widely used to confirm the binding of the peptides to HLA-A*02:01 as its HLA levels can be stabilized by the addition of exogenous HLA-binding peptides but unable to present the endogenous HLA-associated peptides [15,36]. To assess binding strength, we first incubated T2 cells with the WT and mutant peptides, respectively, and then used the W6/32 antibody that targets HLA molecules stabilized by any HLA-binding peptides. The strength of peptide binding between WT and mutant peptides were comparable as suggested by W6/32 staining. Analysis of the pulsed cells by flow cytometry showed that binding of the TP53 (R248 W) mutant peptide to T2 cells was more significant than the background levels of staining to the WT peptide or negative control cells (figure 4), which confirms the specific binding of the TP53 mutant peptide to HLA-A*02:01. Therefore, the R248 W mutation of TP53 can generate a potential tumour-specific neoantigen in the patient with HLA-A*02:01, which can be an ideal target for neoantigen-specific cancer immunotherapy.

## 4. Discussion

TSNAD is a tool for detecting cancer somatic mutations following the best practices of GATK [20]. TSNAD can also provide potential neoantigens [1], which can be either extracellular mutations of membrane proteins or mutant peptides presented by class I MHC molecules. It is critical for biologists without programming background. We applied the antigen-predicting tool of TSNAD to predict neoantigens, including extracellular mutations of membrane proteins and neoantigens presented by MHC class I molecules. And we experimentally verified the specific-binding of the mutated peptide of TP53 we predicted (R248 W, wild-type: GMN**R**RPILTII, mutant: GMN**W**RPILTII) to HLA-A*02:01. The predicted neoantigens in our study were important sources for selecting suitable drug targets. In further study, these predicted neoantigens would need more experimental validation for their potential to be employed as drug targets of T cell or antibody-based immunotherapy.

## Supplementary Material

Supplementary Tables & Figures

## Data accessibility

The software and codes are freely available from https://github.com/jiujiezz/tsnad and the predicted neoantigens are freely available from http://biopharm.zju.edu.cn/lab/database/tsnadb.
