# Supplementary material for: TSNAD: an integrated software for cancer somatic mutation and tumour-specific neoantigen detection
Source: R Soc Open Sci. 2017 Apr 5;4(4):170050. doi: 10.1098/rsos.170050 (PMC5414268; doi:10.1098/rsos.170050)
Supplement: Supplementary Tables & Figures [file rsos170050supp1.pdf]

Royal Society Open Science

Supplementary Material

TSNAD: an Integrated Software for Cancer Somatic Mutation and Tumor-Specific NeoAntigen Detection

Zhan Zhou[1, †], Xingzheng Lyu[2, †], Jingcheng Wu[1], Xiaoyue Yang[1], Shanshan Wu[1], Jie Zhou[1], Xun Gu[3], Zhixi Su[4,*], Shuqing Chen[1,*]

[1]Zhejiang Provincial Key Laboratory of Anti-Cancer Drug Research, College of Pharmaceutical Sciences, Zhejiang University, Hangzhou 310058, China

[2]College of Computer Science and Technology, Zhejiang University, Hangzhou 310013, China

[3]Department of Genetics, Development and Cell Biology, Program of Bioinformatics and Computational Biology, Iowa State University, Ames, IA 50010, USA

[4]State Key Laboratory of Genetic Engineering and MOE Key Laboratory of Contemporary Anthropology, School of Life Sciences, Fudan University, Shanghai 200438, China

[†]These authors have contributed equally to this work

[*]To whom correspondence should be addressed: Zhixi Su: zxsu@fudan.edu.cn and Shuqing Chen: chenshuqing@zju.edu.cn

Supplementary Table S1. The distribution of mutation with the same recurrences in membrane proteins

Mutation recurrences	Number of mutations
1	79198
2	6859
3	1412
4	492
5	180
6	87
7	55
8	25
9	17
10	9
11	7
12	3
13	1
14	1
15	3
16	1
18	1
23	1
25	1
44	1

Supplementary Table S2. The distribution of mutation with the same recurrences in MHC prediction results.

Mutation recurrences	Number of mutations
1	1311238
2	87322
3	14228
4	4063
5	1665
6	792
7	530
8	234
9	173
10	137
11	103
12	57
13	29
14	29
15	42
16	10
17	13
18	11
19	5
20	6
21	5
22	16
23	5
25	7
26	4
27	14
29	2
32	1
35	3
38	11
44	1
57	5
100	4
102	1
106	7
112	1
182	2
200	3
239	5
322	1

Supplementary Table S3. The 65 potential common neoantigens whose corresponding mutations appear in at least 20 out of the 9,155 donors from the ICGC database and had the IC50 less than 500.

Name	HLA_allele	Position	Neoantigen	Mutation	Affinity(nM)	Recurrences
TP53	HLA-A*24:02	9	NWRPILTI	R248W	461	57
TP53	HLA-B*35:01	2	MCNSSCMGSM	G245S	391	38
TP53	HLA-A*11:01	2	GLAPPQHLTR	I195T	380	22
TP53	HLA-A*02:01	7	GMNWRPILTI	R248W	350	57
TP53	HLA-B*35:01	1	YMCNSSCMGSM	G245S	338	38
TP53	HLA-A*02:01	7	GMNWRPILTII	R248W	332	57
TP53	HLA-C*03:04	1	YMCNSSCMGSM	G245S	85	38
TP53	HLA-C*03:03	1	YMCNSSCMGSM	G245S	85	38
SF3B1	HLA-B*40:01	9	QEVRTISALAI	K700E	52	100
SF3B1	HLA-B*40:01	9	QEVRTISAL	K700E	16	100
PTEN	HLA-A*02:01	9	GQTGVMICAYL	R130Q	499	29
PTEN	HLA-C*03:04	5	KAGKGGTGVM	R130G	442	26
PTEN	HLA-C*03:03	5	KAGKGGTGVM	R130G	442	26
PIK3CA	HLA-C*03:04	2	FMKQMNDAL	H1047L	489	27
PIK3CA	HLA-C*03:03	2	FMKQMNDAL	H1047L	489	27
PIK3CA	HLA-C*06:02	9	ARHGGWTTKM	H1047R	457	200
PIK3CA	HLA-A*03:01	0	KAISTRDPLSK	E542K	429	106
PIK3CA	HLA-C*03:04	1	YFMKQMNDAL	H1047L	381	27
PIK3CA	HLA-C*03:03	1	YFMKQMNDAL	H1047L	381	27
PIK3CA	HLA-A*03:01	0	STRDPLSEITK	E545K	321	182
PIK3CA	HLA-C*07:01	9	ARHGGWTTKM	H1047R	249	200
PIK3CA	HLA-A*02:01	2	FMKQMNDAL	H1047L	246	27
PIK3CA	HLA-C*07:02	9	ARHGGWTTKM	H1047R	218	200
PIK3CA	HLA-A*03:01	1	AISTRDPLSK	E542K	213	106
PIK3CA	HLA-A*11:01	2	ISTRDPLSK	E542K	212	106
PIK3CA	HLA-A*03:01	3	STRDPLSK	E542K	203	106
PIK3CA	HLA-A*03:01	10	KTQKVQMK	E726K	161	21
PIK3CA	HLA-A*11:01	9	ALHGGWTTK	H1047L	148	27
PIK3CA	HLA-A*11:01	0	KAISTRDPLSK	E542K	87	106
PIK3CA	HLA-A*11:01	1	AISTRDPLSK	E542K	87	106
PIK3CA	HLA-A*11:01	0	STRDPLSEITK	E545K	81	182
PIK3CA	HLA-A*11:01	3	STRDPLSK	E542K	66	106
PIK3CA	HLA-A*03:01	9	ALHGGWTTK	H1047L	61	27
PIK3CA	HLA-A*11:01	10	KTQKVQMK	E726K	60	21
OPRD1	HLA-C*03:04	2	ASDAYPSAF	C27F	402	25
OPRD1	HLA-C*03:03	2	ASDAYPSAF	C27F	402	25
OPRD1	HLA-A*01:01	2	ASDAYPSAF	C27F	389	25
OPRD1	HLA-C*03:04	1	NASDAYPSAF	C27F	77	25
OPRD1	HLA-C*03:03	1	NASDAYPSAF	C27F	77	25

MUC4	HLA-A*02:01	9	GQATPLPV	H4205Q	228	44
KRAS	HLA-C*03:04	8	GAAGVGKSAL	G12A	217	22
KRAS	HLA-C*03:03	8	GAAGVGKSAL	G12A	217	22
KRAS	HLA-A*02:01	3	KLVVVGADGV	G12D	214	322
KRAS	HLA-C*03:04	8	GAVGVGKSAL	G12V	172	239
KRAS	HLA-C*03:03	8	GAVGVGKSAL	G12V	172	239
KRAS	HLA-A*02:01	3	KLVVVGAV	G12V	163	239
KRAS	HLA-A*02:01	3	KLVVVGAVGV	G12V	112	239
KRAS	HLA-A*02:01	3	KLVVVGACGV	G12C	98	26
KRAS	HLA-B*40:01	0	TEYKLVVVGAV	G12V	90	239
KRAS	HLA-A*02:01	3	KLVVVGAAGV	G12A	82	22
KRAS	HLA-B*07:02	8	GARGVGKSAL	G12R	79	102
GNAS	HLA-B*35:01	10	HVLTSGIF	R844H	199	20
FRG1	HLA-C*03:04	10	QVFQNGKMALL	P140Q	477	22
FRG1	HLA-C*03:03	10	QVFQNGKMALL	P140Q	477	22
FRG1	HLA-C*03:04	10	QVFQNGKMAL	P140Q	180	22
FRG1	HLA-C*03:03	10	QVFQNGKMAL	P140Q	180	22
FAM194B	HLA-A*03:01	10	HLGKEGYLEK	Y139H	413	27
FAM194B	HLA-A*11:01	10	HLGKEGYLEK	Y139H	262	27
CTNNB1	HLA-B*35:01	3	GATTTAPF	S45F	407	20
CTNNB1	HLA-B*35:01	2	SGATTTAPF	S45F	393	20
CTNNB1	HLA-C*07:02	2	SYLDSGIHF	S37F	322	23
CTNNB1	HLA-B*35:01	1	HSGATTTAPF	S45F	99	20
CTNNB1	HLA-A*24:02	2	SYLDSGIHF	S37F	82	23
CHEK2	HLA-B*40:01	9	SEILGETSLM	K416E	49	35
CHEK2	HLA-B*40:01	9	SEILGETSL	K416E	10	35

Supplementary Table S4. The distribution of neoantigens across 20 tumor types

Tumor type	#Neoantigens	#Donors	#Average Neoantigens
Uterus	176130	246	715.98
Skin	410717	584	703.28
Stomach	116429	298	390.70
Lung	74604	224	333.05
Colorectal	140713	443	317.64
Bladder	48348	233	207.50
Cervix	38025	194	196.01
Esophagus	38894	347	112.09
Gall_Bladder	23924	239	100.10
Liver	96283	966	99.67
Ovary	14736	181	81.41
Breast	76006	1072	70.90
Head_and_neck	36381	521	69.83
Kidney	37620	668	56.32
Pancreas	35736	685	52.17
Prostate	18106	499	36.28
Brain	28213	797	35.40
Blood	10166	686	14.82
Bone	2084	164	12.71
Nervous_System	258	108	2.39

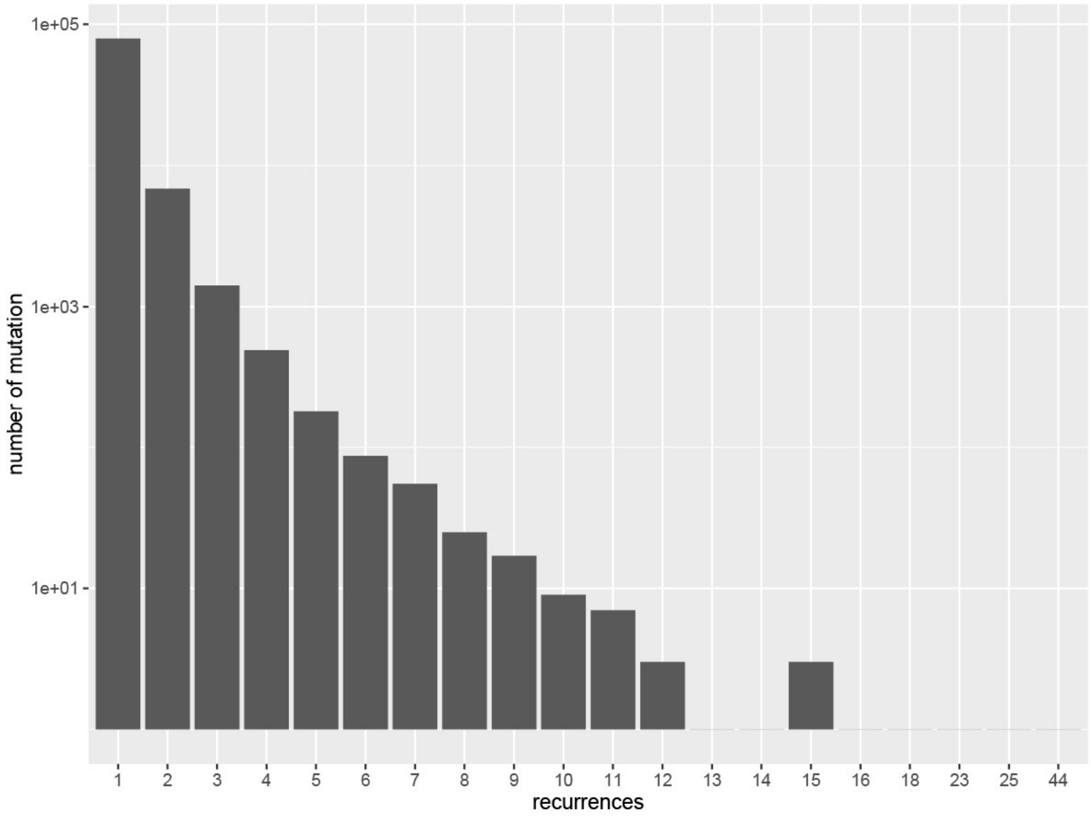

Supplementary Figure S1. The distribution of mutations with the same recurrences in membrane proteins. A majority of these extracellular mutations (89.6%, 79,198 out of 88,354) occur only once in the 9,155 donors.

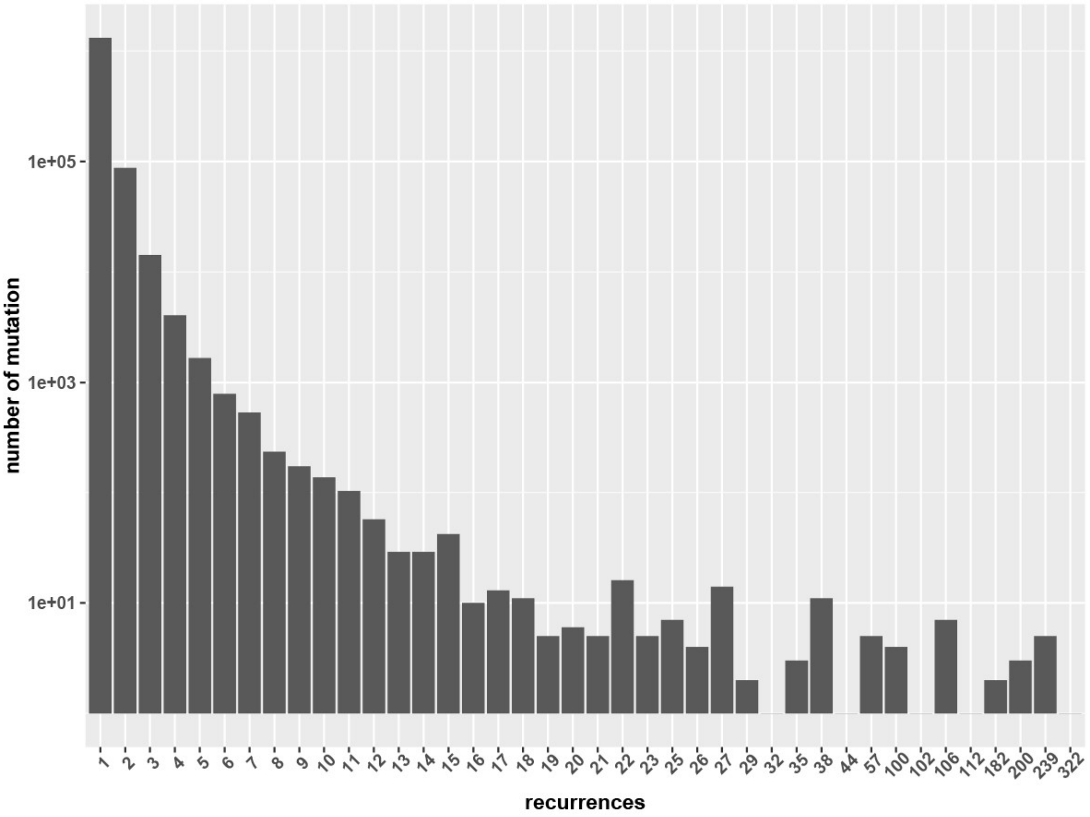

Supplementary Figure S2. The distribution of mutations with the same recurrences in MHC prediction results. 1311238 mutations only occur in one sample.
